# The reliability of non-invasive biophysical outcome measures for evaluating normal and hyperkeratotic foot skin

**DOI:** 10.1186/s13047-015-0083-8

**Published:** 2015-07-09

**Authors:** Farina Hashmi, Ciaran Wright, Christopher Nester, Sharon Lam

**Affiliations:** School of Health Sciences, Centre for Health Sciences Research, University of Salford, Manchester, UK; Postgraduate student, School of Health Sciences, Centre for Health Sciences Research, University of Salford, Manchester, UK; Research lead: Foot and Ankle Research Programme, Centre for Health Sciences Research, School of Health Sciences, University of Salford, Manchester, UK; Senior Innovation Associate, Reckitt Benckiser, Dansom Lane, Hull, UK

**Keywords:** Foot, Skin, Hyperkeratosis, Biomechanics, Physiology, Quantification, Reliability

## Abstract

**Background:**

Hyperkeratosis of foot skin is a common skin problem affecting people of different ages. The clinical presentation of this condition can range from dry flaky skin, which can lead to fissures, to hard callused skin which is often painful and debilitating. The purpose of this study was to test the reliability of certain non-invasive skin measurement devices on foot skin in normal and hyperkeratotic states, with a view to confirming their use as quantitative outcome measures in future clinical trials.

**Methods:**

Twelve healthy adult participants with a range of foot skin conditions (xerotic skin, heel fissures and plantar calluses) were recruited to the study. Measurements of normal and hyperkeratotic skin sites were taken using the following devices: Corneometer® CM 825, Cutometer® 580 MPA, Reviscometer® RVM 600, Visioline® VL 650 Quantiride® and Visioscan® VC 98, by two investigators on two consecutive days. The intra and inter rater reliability and standard error of measurement for each device was calculated.

**Results:**

The data revealed the majority of the devices to be reliable measurement tools for normal and hyperkeratotic foot skin (ICC values > 0.6). The surface evaluation parameters for skin: SEsc and SEsm have greater reliability compared to the SEr measure. The Cutometer® is sensitive to soft tissue movement within the probe, therefore measurement of plantar soft tissue areas should be approached with caution. Reviscometer® measures on callused skin demonstrated an unusually high degree of error.

**Conclusions:**

These results confirm the intra and inter rater reliability of the Corneometer®, Cutometer®, Visioline® and Visioscan® in quantifying specific foot skin biophysical properties.

## Background

Hyperkeratosis of foot skin is a common problem which often manifests as corns, calluses, xerosis or fissured heel skin [1]. These conditions are often unsightly and can be the source of discomfort and pain leading to the deterioration of the quality of life of the individuals involved [2]. The impact of these conditions is even greater in the elderly and people with diabetes where the added complications of peripheral vascular disease place the foot at risk of ulceration, infection and amputation [3 – 5]. In the older population callus can affect balance and consequently increase the risk of falls [6, 7].

Although the causes of these foot dermatoses are multifarious, there are some known aetiologies that can be manipulated to improve the structure and function of the skin (in particular the Stratum Corneum (SC)). Examples of these include altering the hydration of the SC through moisturisation [8 – 10] or removal of the bulk of the hyperkeratotic tissue through keratolytic agents [11 – 14] or via physical debridal. To that end there are many treatments available through professionals and over the counter via pharmacists that purport to remedy these skin problems. Some products appear to have a degree of efficacy, but it is difficult for healthcare practitioners, consumers and product developers to determine which treatments are appropriate for specific types of foot skin conditions. The key barrier is that the majority of the evidence is based on the subjective opinions of the users rather than on objective quantitative measures of skin properties.

In order to evaluate the efficacy of these treatments there is a need for appropriately designed clinical studies that focus on quantifying their effects on specific aspects of foot skin structure and function. Before conducting these clinical trials it is vital to invest time in the selection of instrumentation with the appropriate sensitivity and reliability to measure important changes in foot skin, such as hydration and elastic properties.

Over the last two decades the field of skin science measurement has advanced considerably with the advent of improved technologies, in particular imaging technologies [15 – 22]. The focus has largely been on validating these methods to test various skin sites on the body but never foot skin. The reasons for foot skin being a special case are mainly the unique architecture of plantar skin and the associated problems of image resolution [23]. In the absence of imaging foot skin, the evaluation of the surface architecture, such as texture, could be used in conjunction with the measures already described to provide a comprehensive profile of the condition of the SC.

This paper reports the reliability of four test methods on foot skin. The devices used were the: Corneometer® CM 825, Cutometer® 580 MPA, Reviscometer® RVM 600, Visioscan® VC 98 and Visioline® VL 650 Quantiride® (Courage-Khazaka, Cologne, Germany). Intra and intertester reliability is reported along with Standard Error of Measurement (SEM) data.

## Methods

The study protocol was reviewed and approved by the University of Salford, Department of Health Sciences Ethical Approval Committee (HSCR12/55). An intrarater and interrater design was applied. Each participant attended for two consecutive appointments. At each occasion both raters took measurements from the participant. Intrarater reliability was evaluated by comparing the measurements taken by the same rater two days apart. Interrater reliability was evaluated by comparing measurements taken by the first (Investigator A) and second (Investigator B) rater during the same session (Day 1 and Day 2).

### Participants

A total of 12 healthy participants took part in the study. All 12 participants had measurements taken from two standardised (control) normal skin sites: plantar metatarsal area (PMA) and the plantar aspect of the base of the fifth metatarsal (5th met. base). These sites were chosen because it was assumed that the mechanical properties of the skin varied between these locations due to the different degrees of weight bearing that they are ordinarily subjected to. There were 7 people with forefoot plantar callus of which measurements were taken from the callus plaque and the adjacent non callused skin (PMA). The remaining 5 participants had heel fissures and measurements were taken from the centre of one fissure and the plantar aspect of the heels where the skin was xerotic.

#### Inclusion criteria

All participants were assessed for the presence of plantar forefoot callus or closed heel fissures. The identification of these conditions were achieved using clinical assessment criteria currently used in practice [24].

#### Exclusion criteria

Participants were excluded from the study if they presented with current general skin disorders affecting the foot such as dermatitis, psoriasis, un-healed skin wounds, ulcers or blisters. Open heel fissures were also excluded. Any participant with a known systematic disease including peripheral vascular disease or musculoskeletal disorders of the foot or ankle, rheumatoid arthritis or diabetes was excluded. Participants were asked not to use any foot products (e.g. creams and powders) in the 48 hours leading up to their appointment. If they were unable to stop using a product due to medical or personal reasons, they were excluded from the study.

### Preparation of the foot and marking of the skin measurement sites

Prior to performing measurements participants acclimatised to room temperature and humidity conditions for at least 15 minutes. The participant sat on a plinth with legs extended and the plantar aspects of both feet facing the investigator. If the participant found it difficult to keep the foot still during testing, an adapted leg and ankle brace was applied to the leg.

A digital photograph of the heel fissure or callus before and after marking of the skin was taken and inserted in the case report form (CRF). These images acted as a guide for the position of the index lesion.

#### Marking of the centre of the heel fissure

**Fig. 1:**
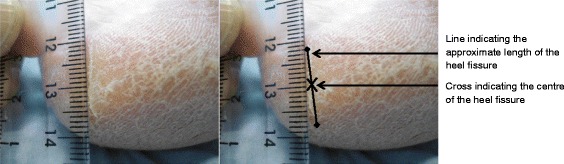
Heel fissure images. The first image is a view of the heel fissure before marking and the second is a view captured during length measurement and marking of the centre of the fissure NB: The line drawn on this diagram is used to describe how the centre of the heel fissure was identified. During testing a single dot or small cross was marked on the area to indicate the centre of the fissure

The length of the heel crack was measured using a ruler and the centre of the fissure was marked using a water soluble pen (Fig. 1). In order to ensure that repeated measures could be taken at the same skin site on consecutive days, an area of skin on the plantar aspect of the heel was selected and also marked to act as a reference point. This reference point was also used as a measurement site, i.e. xerotic plantar heel skin.

#### Marking of the centre of plantar callus

**Fig. 2:**
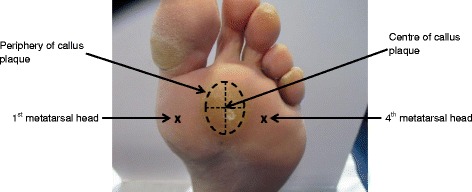
Marking of the centre of plantar forefoot callus. The line drawn on this diagram is used to describe how the centre of the callus was identified. During testing a single dot or small cross was marked on the area to indicate the centre of the callus

Using a ruler the callus plaque was bisected along the horizontal and vertical axes and the centre was marked (Fig. 2). A region of skin on the PMA that had no callus, i.e. normal skin, was selected and marked. This site was either the 1st or 4th metatarsal head depending on the location of the plantar callus plaque. This was achieved by palpating the head of the metatarsal and marking the site.

#### Second normal skin site

A second site in the region of the plantar aspect of the 5th metatarsal base was selected as another normal skin site.

During the measurements, the participants sat in a relaxed position, generating the least possible muscle activity. Each rater performed consecutive measurements on each location.

### Skin surface hydration measurement

The Corneometer® CM 825 (Courage-Khazaka, Cologne, Germany) was used to measure skin surface hydration. The measuring principle is based on a validated - capacitance method [25]. The probe (surface area of 0.95 cm^2^) contains an interdigital grid of gold electrodes, covered by a low dielectric vitrified material of 20 μm thickness. The grids are 50 μm wide with interdigital spacing of 75 μm. A resonating system in the instrument detects the frequency shift of the oscillating system related to the capacitance (and hence hydration) of the tissue in contact with the probe. The frequency shifts from 0.95 MHz for a hydrated medium to 1.15 MHz for a dry medium. The capacity is measured within 1 to 1.5 s of application of the probe on the skin. The variable total capacitance of the skin is converted to arbitrary units (AU) of skin surface hydration. The range of the device is 1 to 120 AU. To allow a constant pressure (1 N ± 10 %) of the probe on the skin surface a spring system is incorporated and the end of the probe displaced 2 mm when placed in contact with the skin [26]. Ten repeated measures were taken at each skin site and an average value calculated.

### Skin elasticity measurement

The Cutometer® 580 MPA (Courage-Khazaka, Cologne, Germany) was used to measure skin elasticity as it is widely regarded as the ‘gold standard’. The reliability of the Cutometer has been successfully tested on normal skin, where the best reproducibility was obtained for the maximum distension of the skin [27]. This method has been tested for reliability (CV: 0.3 – 6.3 %) and used successfully on foot skin in patients without callus or other skin pathologies [28]. The central suction aperture was 8 mm in diameter. One time/strain cycle was used, consisting of traction under negative pressure for 30 s at, 500 mbar, followed by release and data recording for a further 30 s. For the purpose of this study the Uf (maximum distension) value was used for analysis. One measurement was taken at each skin site.

### Collagen and elastin fibre organisation measurement

The Reviscometer® RVM 600 (Courage-Khazaka, Cologne, Germany) probe contains two stylus sensors, which are 2 mm apart. When placed onto the skin, one sensor transmits an acoustic shock wave of 1.77 mJ to the recipient sensor. The time this wave travels from one sensor to the other is called the resonance running time (RRT). A low RRT corresponds to a more parallel alignment of fibres. The wave penetration depth is between 0.5 and 0.7 mm [29]. Measurements were performed in one direction only and 20 repeated measures were taken and a mean RRT calculated.

### Surface evaluation of living skin (SELs)

The SELs parameters smoothness (SEsm), roughness (SEr) and scaliness (SEsc) were determined by the Visioscan® VC 98 and the software SELS (Courage-Khazaka, Cologne, Germany). The Visioscan® VC 98 has two lights arranged on opposite sides of the camera in order to illuminate the skin uniformly. The spectrum of light, its intensity and the way it is arranged are designed into the device so that only the stratum corneum is detected and measured, without reflections from deeper skin layers [30]. The skin area measured by the camera is 6 × 8 mm. The device was calibrated according to the manufacturer’s instructions prior to commencing the study. The camera was placed on the marked areas of skin, images captured, saved and processed. A resolution of 768 × 576 × 32b (RGB 32) was used. According to the recommendations of the manufacturer the calculation ‘area 2’ was selected for all calculations.

### Heel fissure depth measurement

The skin replica analysis, which is a quantitative method for assessment of micro-topographic features, has been previously described [31]. For heel skin an adapted version of this method was used. Briefly, negative skin replicas were obtained with Silflo from fissured heel skin. A thin layer (approximately 2 to 3 mm thick) of freshly prepared Silflo silicone was gently spread over the region of skin and a piece of paper was applied to the Silflo whilst still wet and then allowed to polymerize. This occurred within 5 min, and subsequently the paper together with the replica was lifted from the skin. Each specimen was coded and stored in an individual envelope. The same silicone rubber materials, catalyst and procedure were used for all subjects. The replicas were analysed using a specific wrinkle analysis apparatus designed to measure facial wrinkles Visioline® VL 650 Quantiride® (Courage-Khazaka, Cologne, Germany). Each replica was placed horizontally on the microscope platform and illuminated with an oblique incident light (35°) by means of optic fibres, thus casting a measureable shadow behind the crest (negative furrow made by the fissure). The software accompanying the device applies the following equation in order to quantify the depth of the fissure: F = d * tanα, where F = fissure, d = shadow and α = 35° angle (fixed by light source).

Arbitrary units are used by the Corneometer® and the Visioscan®. For these instruments there is no direct measure of a physiological quantity, rather a signal is measured that is affected by a biophysical parameter (e.g. water content, uniformity of SC) which is associated with a biophysical property (e.g. hydration or texture).

### Measurement of room temperature and humidity

Relative humidity (RH) and room temperature were monitored and recorded during the test procedures on day one and day two, via sensors incorporated in each of the devices. Temperature and RH values were recorded each time a measurement was taken using the probes. The average values were calculated for each day and compared using an independent sample *T*-test to test for day-to-day differences in environmental conditions.

### Statistical analysis

For assessing intra and interrater reliability Intraclass Correlation Coefficient agreement [ICC (2,1)] with 95 % Confidence Intervals (CI) were calculated as reliability estimates of all obtained values as recommended by the COnsensus – based Standards for the selection of health Measurement INstruments (COSMIN) [32, 33]. ICC is preferred as it takes into account the systematic and random errors [32]. Bland – Altman’s limits of agreement (95 % LOA), and mean difference (or bias) were used for evaluating agreement between measures [34]. Measurement errors were estimated by calculating the Standard Error of Measurement (SEM) using the formula: SEM consistency = SD difference/√2, where SD difference = SD of the mean differences between Investigator A and B or between Day 1 and 2.

An ICC of 0.7 was assumed as the minimal level of reliability. Criteria by Landis and Koch (1977) were used to interpret the ICC agreement values: *slight* (r = 0.00 – 0.19), *fair* (r = 0.20 – 0.39); *moderate* (r = 0.40 – 0.59); *substantial* (r = 0.60 – 0.79); and *almost perfect* (r = 0.80 – 1.0) reliability [35].

The Wilcoxan Test was used to test for differences in biophysical parameters between different skin types. These differences were compared to the SEM values to ascertain whether they lay within or outside the boundaries of error for each method, therefore confirming whether the differences were due to the skin type or due to errors in the methods. All statistical tests were performed using a two-tailed 5 % overall significance level.

Data was analysed using SPSS version 20.0 (IBM®, SPSS, Statistics) and GraphPad PRISM® version 6.04 (GraphPad PRISM Software Ltd).

## Results

A total of 12 healthy, adult, Caucasian volunteers participated in the study (4 males and 8 females). The mean age was 44 ± 14 years. The descriptive characteristics of the participants are summarised in table 1. The average environmental temperature was 23.6 ± 1.3 °C (day 1) and 22.9 ± 0.7 °C (day 2). The average RH was 52.7 ± 6.7 % (day 1) and 53.6 ± 9.7 % (day 2). There was no statistically significant difference in temperature (p = 0.84) and RH (p = 0.89) between day one and day two.

**Table 1:** Selected scaliness parameters, hydration, elasticity, collagen & elastin fibre organisation and fissure depth measures testing intra – rater reliability

					Bland - Altman data	
		ICC agreement	95 % CI	SEM consistency	Mean difference [SD]	95 % LOA
**Investigator A**						
**Callus**	**Hydration [AU]**	0.61	−0.95 - 0.93	0.26	−1.25 [3.11]	−7.30 – 4.80
	**Elasticity [mm]**	0.77	−0.17 - 0.97	0.06	0.30 [0.40]	−0.47 – 1.08
	**Collagen and elastin fibre organisation [AU]**	0.98	0.88 - 1.00	6.76	1.69 [44.50]	−85.50 – 88.92
	**SEsm [AU]**	0.88	0.34 – 0.98	1.23	−0.61 [24.76]	−49.13 – 47.91
	**SEr [AU]**	0.84	0.10 – 0.97	0.29	−0.10 [1.74]	−3.51 – 3.31
	**SEsc [AU]**	0.38	0.84 – 1.51	0.03	−0.20 [0.38]	−0.94 – 0.55
**Heel fissure**	**Hydration [AU]**	0.93	0.26 – 0.99	0.35	0.26 [2.33]	−4.30 – 4.82
	**Elasticity [mm]**	0.82	−4.42 – 1.00	0.00	0.03 [0.10]	−0.16 – 0.21
	**SEsm [AU]**	1.00	8.85 – 0.90	0.00	−11.35 [33.12]	−76.27 – 53.57
	**SEr [AU]**	0.38	0.97 – 6.14	1.24	−0.20 [2.47]	−5.03 – 4.63
	**SEsc [AU]**	0.73	0.98 – 21.89	0.04	−0.07 [0.12]	−0.29 – 0.16
	**Fissure depth [**μ**m]**	0.95	0.46 – 1.00	4.83	−20.08 [29.74]	−78.37 – 38.21
**Xerotic plantar heel skin**	**Hydration [AU]**	0.88	−0.49 – 1.00	0.37	0.59 [2.05]	−3.43 – 4.61
	**Elasticity [mm]**	0.53	−9.25 – 0.97	0.01	−0.05 [0.11]	−0.25 – 0.16
	**Collagen and elastin fibre organisation [AU]**	0.98	0.33 – 1.00	0.51	−12.58 [8.37]	−28.98 – 3.83
	**SEsm [AU]**	0.51	0.46 – 0.96	14.13	−27.07 [20.70]	−67.64 – 13.51
	**SEr [AU]**	0.68	0.82 – 2.17	0.17	−0.71 [0.84]	−2.35 – 0.94
	**SEsc [AU]**	0.31	0.93 – 7.56	0.41	0.33 [0.59]	−0.82 – 1.47
**PMA**	**Hydration [AU]**	0.97	0.86 – 1.00	0.16	−0.35 [2.64]	−5.50 – 4.82
	**Elasticity [mm]**	0.45	−2.68 – 0.92	7.9 x10^−5^	0.08 [0.19]	−0.31 – 0.46
	**Collagen and elastin fibre organisation [AU]**	0.88	0.32 – 0.98	32.02	18.06 [95.33]	−168.80 – 204.90
	**SEsm [AU]**	0.96	0.84 – 0.99	0.07	−0.61 [10.65]	−21.48 – 20.26
	**SEr [AU]**	0.76	0.06 – 0.94	0.48	−0.24 [2.06]	−4.28 – 3.79
	**SEsc [AU]**	0.78	0.14 – 0.94	0.08	−0.07 [0.64]	−1.32 – 1.18
**5th met. base**	**Hydration [AU]**	0.92	0.73 – 0.98	0.15	−1.10 [2.77]	−6.51 – 4.32
	**Elasticity [mm]**	0.80	0.11 – 0.95	0.34	−0.02 [0.16]	−0.33 – 0.30
	**Collagen and elastin fibre organisation [AU]**	0.98	0.92 – 1.00	4.16	1.61 [36.50]	−69.92 – 73.14
	**SEsm [AU]**	0.72	0.24 – 0.90	0.44	−4.18 [22.91]	−49.09 – 40.73
	**SEr [AU]**	0.66	0.03 – 0.88	0.31	0.11 [1.16]	−2.17 – 2.38
	**SEsc [AU]**	0.72	0.23 – 0.90	0.49	0.17 [1.21]	−2.20 – 2.53
					**Bland – Altman data**	
		**ICC agreement**	**95 % CI**	**SEM consistency**	**Mean difference [SD]**	**95 % LOA**
**Investigator B**						
**Callus**	**Hydration [AU]**	0.40	−3.17 – 0.90	0.73	−0.99 [3.67]	−8.18 – 6.19
	**Elasticity [mm]**	0.98	0.82 – 1.00	0.02	0.01 [0.17]	−0.34 – 0.35
	**Collagen and elastin fibre organisation [AU]**	1.00	0.92 – 1.00	5.62	18.87 [46.12]	−71.54 – 109.30
	**SEsm [AU]**	0.82	0.11 – 0.96	0.34	4.97 [22.52]	−39.17 – 49.11
	**SEr [AU]**	0.84	0.19 – 0.97	0.43	0.40 [1.99]	−3.50 – 4.30
	**SEsc [AU]**	0.89		0.02	−0.15 [0.24]	−0.61 – 0.31
**Heel fissure**	**Hydration [AU]**	0.89	−0.28 – 1.00	0.94	0.29 [3.21]	−6.01 – 6.60
	**Elasticity [mm]**	0.95	−0.05 – 1.00	0.00	−0.06 [0.02]	−0.10 – 0.01
	**SEsm [AU]**	0.68	0.94 – 5.03	12.62	4.97 [22.52]	−39.17 – 49.11
	**SEr [AU]**	0.67	0.98 – 32.45	1.21	0.44 [2.29]	−4.05 – 4.94
	**SEsc [AU]**	0.61	0.98 – 20.97	0.02	0.06 [0.17]	−0.28 – 0.39
	**Fissure depth [**μ**m]**	0.64	0.47 – 0.93	49.90	5.56 [14.06]	−270.10 – 281.20
**Xerotic plantar heel skin**	**Hydration [AU]**	0.84	−0.52 – 0.98	0.64	0.68 [2.40]	−4.03 – 5.38
	**Elasticity [mm]**	0.40	7.74 – 0.96	0.01	−0.18 [0.07]	−0.15 – 0.12
	**Collagen and elastin fibre organisation [AU]**	0.86	−0.19 – 0.99	6.55	−16.98 [19.34]	−54.88 – 20.93
	**SEsm [AU]**	0.76	0.71 – 0.98	8.04	−15.60 [21.64]	−58.01 – 26.82
	**SEr [AU]**	0.37	0.66 – 0.94	0.05	−0.64 [0.58]	−1.77 – 0.49
	**SEsc [AU]**	0.86	0.22 – 0.99	0.06	0.11 [0.19]	−0.26 – 0.48
**PMA**	**Hydration [AU]**	1.00	0.89 – 1.00	0.00	−1.43 [1.92]	−5.20 – 2.33
	**Elasticity [mm]**	0.55	−6.05 – 0.94	0.04	0.00 [0.15]	−3.00 – 0.30
	**Collagen and elastin fibre organisation [AU]**	0.96	0.75 – 0.99	5.42	−5.96 [37.55]	−79.56 – 67.64
	**SEsm [AU]**	0.95	0.81 – 0.99	1.46	5.33 [11.50]	−17.21 – 27.87
	**SEr [AU]**	0.89	0.58 – 0.97	0.09	−0.05 [1.04]	−2.10 – 1.98
	**SEsc [AU]**	0.89	0.59 – 0.97	0.28	−0.21 [0.42]	−1.02 – 0.61
**5th met. base**	**Hydration [AU]**	0.91	0.72 – 0.98	0.16	−1.20 [3.90]	−8.85 – 6.45
	**Elasticity [mm]**	0.54	−0.45 – 0.88	0.03	0.05 [0.10]	−0.14 – 0.25
	**Collagen and elastin fibre organisation [AU]**	0.99	0.95 – 1.00	3.17	−65.61 [88.91]	−65.61 – 88.91
	**SEsm [AU]**	0.92	0.78 – 0.97	2.93	−4.16 [11.29]	−26.30 – 17.97
	**SEr [AU]**	0.84	0.52 – 0.94	0.12	0.51 [0.90]	−1.26 – 2.28
	**SEsc [AU]**	0.88	0.67 – 0.96	0.07	0.03 [0.48]	−0.91 – 0.96

Summarised statistics for intrarater reliability are presented for each of the skin tests conducted by investigator A and B in table 1. Overall intrarater reliability ranged from *fair* to *perfect* with ICC values between 0.31 and 1.00. Table 2 summarises the interrater reliability statistics. The interrater reliability also ranged from *fair* to *perfect* with ICC values between 0.29 and 1.00.

**Table 2:** Selected scaliness parameters, hydration, elasticity, collagen & elastin fibre organisation and fissure depth measures testing inter - rater reliability

					Bland - Altman data	
		ICC agreement	95 % CI	SEM consistency	Mean difference [SD]	95 % LOA
**Day 1**						
**Callus**	**Hydration [AU]**	0.89	0.30 – 0.98	0.08	−0.14 [2.20]	−4.45 – 4.18
	**Elasticity [mm]**	0.83	−0.01 – 0.98	0.01	0.31 [0.37]	−0.41 – 1.03
	**Collagen and elastin fibre organisation [AU]**	1.00	0.74 – 0.99	0.00	−45.10 [66.09]	−174.6 – 84.44
	**SEsm [AU]**	0.91	0.59 – 0.98	1.54	−5.42 [18.15]	−41.00 – 30.16
	**SEr [AU]**	0.62	0.93 – 1.23	0.32	−0.47 [3.03]	−6.41 – 5.46
	**SEsc [AU]**	0.87	0.31 – 0.97	0.02	−0.02 [0.20]	−0.42 – 0.38
**Heel fissure**	**Hydration [AU]**	0.98	0.82 – 1.00	0.17	−0.77 [1.64]	−3.97 – 2.44
	**Elasticity [mm]**	0.78	−0.34 – 0.98	0.01	0.11 [0.08]	−0.05 – 0.27
	**SEsm [AU]**	0.92	0.24 – 1.00	0.30	−9.83 [12.66]	−34.63 – 4.98
	**SEr [AU]**	0.90	0.01 – 0.99	0.16	−0.41 [0.76]	−1.89 – 1.08
	**SEsc [AU]**	0.74	0.67 – 0.98	0.03	−0.08 [0.10]	−0.28 – 0.12
	**Fissure depth [** **μm]**	0.33	−4.50 – 0.95	0.49	−67.20 [120.10]	−302.80 – 168.20
**Xerotic plantar heel skin**	**Hydration [AU]**	0.97	0.79 – 1.00	0.05	−0.40 [1.23]	−2.81 – 2.00
	**Elasticity [mm]**	0.86	−0.32 – 0.99	0.02	0.03 [0.06]	−0.08 – 0.14
	**Collagen and elastin fibre organisation [AU]**	0.95	0.26 – 1.00	3.05	16.05 [14.68]	−12.71 – 44.81
	**SEsm [AU]**	0.96	0.03 – 1.00	0.68	−12.01 [6.53]	−24.82 – 0.79
	**SEr [AU]**	0.29	0.96 – 9.93	0.12	−0.19 [0.73]	−1.62 – 1.25
	**SEsc [AU]**	0.83	0.56 – 0.99	0.10	0.20 [0.36]	−0.51 – 0.91
**PMA**	**Hydration [AU]**	1.00	0.97 – 1.00	0.00	−0.48 [1.26]	−2.95 – 1.99
	**Elasticity [mm]**	0.69	−0.19 – 0.95	0.00	0.19 [0.09]	0.01 – 0.36
	**Collagen and elastin fibre organisation [AU]**	0.90	0.48 – 0.98	25.46	38.30 [87.57]	−133.30 – 209.90
	**SEsm [AU]**	0.97	0.70 – 0.99	0.92	−7.34 [7.68]	−22.39 – 7.71
	**SEr [AU]**	0.86	0.46 – 0.96	0.37	−0.09 [1.65]	−3.31 – 3.14
	**SEsc [AU]**	0.84	0.44 – 0.96	0.04	0.10 [0.44]	−0.76 – 0.96
**5th met. base**	**Hydration [AU]**	0.97	0.88 – 0.99	0.21	−0.89 [2.18]	−5.16 – 3.38
	**Elasticity [mm]**	0.74	0.00 – 0.93	0.06	0.11 [0.14]	−0.17 – 0.39
	**Collagen and elastin fibre organisation [AU]**	0.96	0.84 – 0.99	8.83	−31.32 [58.35]	−145.70 – 83.04
	**SEsm [AU]**	0.82	0.50 – 0.94	1.74	0.19 [17.45]	−34.02 – 34.40
	**SEr [AU]**	0.65	0.07 – 0.87	0.37	−0.34 [1.21]	−2.70 – 2.03
	**SEsc [AU]**	0.84	0.58 – 0.94	0.34	0.29 [0.94]	−1.56 – 2.14
					**Bland – Altman data**	
		**ICC agreement**	**95 % CI**	**SEM consistency**	**Mean difference [SD]**	**95 % LOA**
**Day 2**						
**Callus**	**Hydration [AU]**	0.97	0.82 – 1.00	0.05	0.12 [0.97]	−1.78 – 2.02
	**Elasticity [mm]**	0.99	0.99 – 1.00	0.00	0.02 [0.02]	−0.02 – 0.05
	**Collagen and elastin fibre organisation [AU]**	0.97	0.82 – 0.99	9.51	−27.92 [51.18]	−128.2 - -72.38
	**SEsm [AU]**	0.94	0.67 – 0.99	9.47	0.16 [17.01]	−33.19 – 33.51
	**SEr [AU]**	0.98	0.88 – 1.00	0.18	0.03 [0.63]	−1.21 – 1.26
	**SEsc [AU]**	0.86	0.26 – 0.97	0.01	0.02 [0.20]	−0.37 – 0.41
**Heel fissure**	**Hydration [AU]**	0.97	0.72 – 1.00	0.06	−0.74 [1.07]	−2.80 – 1.37
	**Elasticity [mm]**	0.89	−0.42 – 0.99	0.00	0.03 [0.08]	−0.12 – 0.18
	**SEsm [AU]**	0.78	0.99 – 102.52	5.48	−0.45 [6.92]	−14.01 – 13.11
	**SEr [AU]**	0.99	0.93 – 1.00	0.14	0.24 [0.41]	−0.57 – 1.04
	**SEsc [AU]**	0.95	0.10 – 1.00	0.01	−0.01 [0.08]	−0.17 – 0.15
	**Fissure depth [** **μm]**	0.82	−0.35 – 0.99	13.71	−41.65 [44.17]	−128.20 – 44.92
**Xerotic plantar heel skin**	**Hydration [AU]**	0.95	0.57 – 1.00	0.06	−0.32 [1.09]	−2.46 – 1.82
	**Elasticity [mm]**	0.69	−0.51 – 0.98	0.02	0.06 [0.06]	−0.05 – 0.17
	**Collagen and elastin fibre organisation [AU]**	0.85	−0.97 – 0.99	10.66	11.65 [29.32]	−45.82 –69.12
	**SEsm [AU]**	0.91	0.99 – 1.26	7.15	−0.54 [8.75]	−17.70 – 16.61
	**SEr [AU]**	0.76	0.98 – 4.99	0.21	−0.12 [0.41]	−0.93 – 0.68
	**SEsc [AU]**	0.75	0.99 – 27.38	0.10	−0.01 [0.14]	−0.29 – 0.27
**PMA**	**Hydration [AU]**	0.97	0.83 – 1.00	0.37	−1.56 [2.80]	−7.05 – 3.93
	**Elasticity [mm]**	0.70	−0.34 – 0.95	0.04	0.11 [0.11]	−0.10 – 0.32
	**Collagen and elastin fibre organisation [AU]**	0.96	0.79 – 0.99	3.03	14.29 [31.34]	47.15 –75.72
	**SEsm [AU]**	0.99	0.97 – 1.00	0.92	−1.41 [4.17]	−9.58 – 6.77
	**SEr [AU]**	0.91	0.67 – 0.98	0.28	0.11 [0.94]	−1.74 – 1.95
	**SEsc [AU]**	0.99	0.97 – 1.00	0.01	−0.03 [0.15]	−0.33 – 0.27
**5th met. base**	**Hydration [AU]**	0.93	0.77 – 0.98	0.61	−1.00 [3.11]	−7.09 – 5.09
	**Elasticity [mm]**	0.38	−0.22 – 0.80	0.05	0.18 [0.10]	−0.01 – 0.38
	**Collagen and elastin fibre organisation [AU]**	0.97	0.87 – 0.99	5.70	−21.28 [43.27]	−1.06.10 – 63.52
	**SEsm [AU]**	0.92	0.79 – 0.97	2.01	0.21 [13.05]	−25.38 – 25.80
	**SEr [AU]**	0.71	0.19 – 1.00	0.20	0.07 [1.22]	−2.30 – 2.47
	**SEsc [AU]**	0.93	0.80 – 1.00	0.13	0.15 [0.32]	−0.48 – 0.78

### Hydration measures using the Corneometer® CM 825

The *intrarater reliability* values lay between 0.88 and 1 (*almost perfect*) in all cases except for the two measures taken from callused skin, where the readings were 0.61 (95 % CI [−0.95 - 0.93]) and 0.40 (95 % CI [−3.17 – 0.90]) for Investigator A and B, respectively. Bland – Altman plots revealed that the greater part of the differences between the investigators was less than 1 AU for the callused skin. The overall SEM ranged from 0.00 AU (PMA) to 0.94 AU (heel fissure).

The *interrater reliability* was above 0.89 (*almost perfect*) in all cases. The SEM ranged from 0.00 AU (PMA, day 1) to 0.61 AU (5th met. base).

#### Elasticity measures using Cutometer® 580 MPA

The *intrarater reliability* data showed the majority of the ICC values being above 0.70, ranging from 0.77 to 0.89. The five values that were below this range were of *moderate* reliability (ICC: 0.40 – 0.55) and were for the plantar heel and PMA skin sites. Bland – Altman plots revealed that the greater part of the differences between the investigators was less than 0.41 mm. Measurement errors expressed as SEM ranged from 0.00 mm (heel fissure) to 0.34 mm (5th met base).

In terms of *interrater* reliability all ICC values were above 0.60 except for one value classed as *fair*: 0.23 (5th met. base, Day 2, 95 % CI [−0.22, 0.80]). Bland – Altman plots data showed the greater part of the differences between the days was less than 0.36 mm for the plantar heel skin, PMA and 5th met. base. SEM values ranged from 0.00 mm (PMA, day1; callus, day 2; heel fissure, day 2) to 0.06 mm (5th met. base).

#### Collagen and elastin fibre organisation using the Reviscometer® RVM 600

All (except one) of the *intrarater reliability* data was classed as *almost perfect* with ICC values between 0.86 (95 % CI [−0.19, 0.99] to 1.00 (95 % CI [0.92 – 1.00]). Measurement errors expressed as SEM ranged from 0.51 AU (plantar heel) to 6.76 AU (callus) with one outlier of 32.02 AU (PMA, Investigator A).

In terms of *interrater* reliability all ICC values were classes as *almost perfect* (0.85 – 1.00). Measurement errors expressed as SEM ranged from 22.17 AU to 61.92 AU for all skin sites except for callused skin where values of 89.05 AU (Day 1) and 324.06 AU (Day 2).

#### Surface evaluation of living skin (SELs) parameters: smoothness (SEsm), roughness (SEr) and scaliness (SEsc) using the Visioscan® VC 98 and the software SELS

From the *intrarater reliability* analysis, the data ranged from being *fair* to *almost perfect* with reading of between 0.31 and 0.96. The majority of the reliability values were all above 0.6. The majority of the reliability values that lay in the 0.4 to 0.6 range were from the parameter SEsc and those values below 0.4 were from the SEr parameter. The values of SEM ranged between 0.00 and 17.51 AU.

The *interrater reliability* values were better in that the range varied from 0.62 to 0.99 (*moderate* to *almost perfect*) for all except one value (0.29 for parameter SEr). The SEM values ranged from 0.06 to 12.84 AU.

#### Heel fissure depth measurement

Intrarater reliability for Investigator A was 0.95 (95 % CI [0.46, 1.00]) and for Investigator B 0.64 (95 % CI [0.47, 0.93]). Interrater reliability on Day 1 was 0.33 (95 % CI [−4.50, 0.95]) and on Day 2 an ICC of 0.82 (95 % CI [−0.35, 0.9]). The data for the ICC value of 0.20 when plotted on a Bland – Altman graph showed the mean difference between investigators to be −67.20 ± 120.10 μm, which lies within the LOA (−302.8 to 168.20 μm). The maximum value for the SEM was 84.96 μm.

#### Comparison of measurements from each skin site

**Fig. 3:**
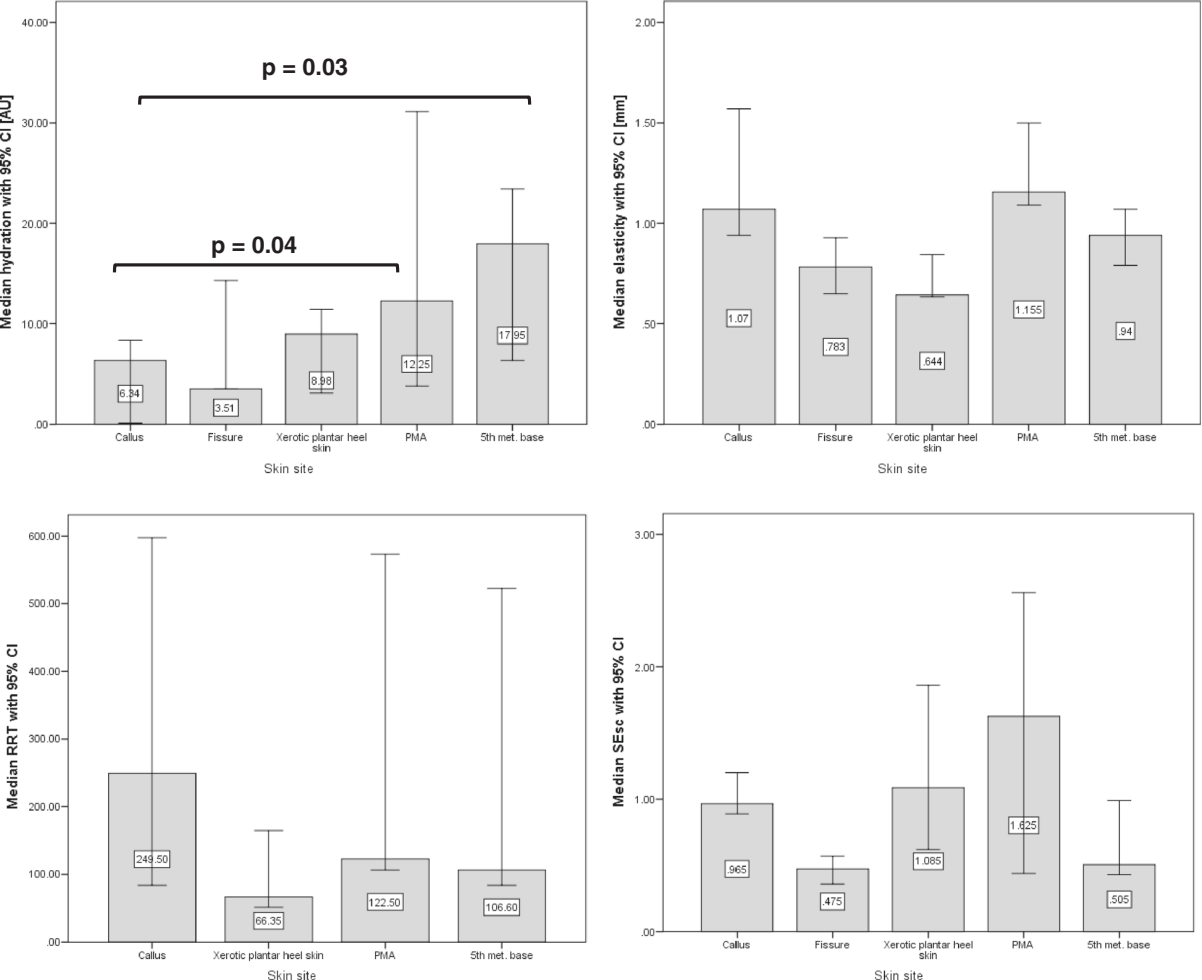
Median (95 % CI) hydration, elasticity, RRT and SEsc for all skin sites. Measurements taken by Investigator A

**Fig. 4:**
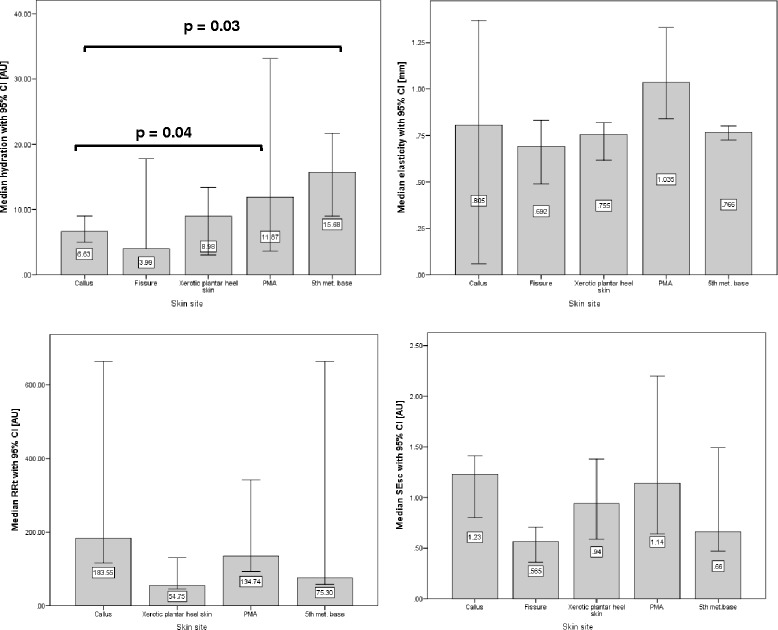
Median (95 % CI) hydration, elasticity, RRT and SEsc for all skin sites. Measurements taken by Investigator B

Post hoc analyses were performed to compare the measures taken from different skin sites (Figs. 3 and 4). The only statistically significant differences found were between PMA and 5th met. base sites compared to callused skin hydration readings (p = 0.04 and 0.03, respectively). These statistical differences were found in the data for both investigators.

The minimum difference in hydration between sites was 1.37 AU (callus v fissure). The differences in elasticity that lie below the SEM for the device are 0.29 mm (PMA v 5th met. base) and 0.10 mm (fissure v xerotic plantar heel skin). The only RRT below the SEM for the Reviscometer was 19.32 AU (fissure v 5th met. base). For the SELS measure SEsc, there were two differences below the SEM: 0.16 AU (fissure v xerotic plantar heel skin) and 0.04 (fissure v 5th met. base).

## Discussion

This study investigated the reliability of four skin measurement devices using the checklist outlined by the COSMIN study [33]. Generally, all test data demonstrated consistent and good reliability for both intra – rater and inter – rater measures. The only two measurement methods that showed consistent and low reliability were the SEr outcome (for the majority of skin sites) and the elasticity measurement of plantar heel and PMA skin sites. There were also unusually high error values for the reviscometer data for the callused skin sites only.

The SELS method is based on the graphic depiction of the living skin under specific illumination and the electronic processing and evaluation of the resulting image. We tested three parameters with this method: skin smoothness (SEsm) which is calculated by averaging the width and depth of wrinkles or in the case of foot skin the width and depth of skin striations; skin roughness (SEr) depicted by the proportion of grey pixels (above a threshold level) compared to the whole image and the peak to peak distances of those pixels; and scaliness (SEsc) which portrays the level of dryness of the SC by calculating the proportion of bright pixels compared to the whole image [30]. These approaches to calculating skin topography have been designed to measure skin other than plantar skin, so it is important to gain an understanding of what is specifically being measured from images captured from plantar skin in different states. The clinical signs of xerotic foot skin and plantar callus are easily distinguished. The classic appearance of xerotic heel skin is diffuse scaliness, i.e. abnormally dry SC cells in the process of desquamation. Plantar callus has a degree of scale on the surface however the plaque itself will consist of impacted, indurated SC tissue which can sometimes appear smooth on the surface. Although in both cases the skin surface may appear uneven it may be that the sensitivity of the SEr measure is not suitable for measuring small changes in surface texture of callused skin. As the development of skin scales is more prominent in foot skin it may be that SEsc provides a more realistic measure of the surface texture of the skin. The SEsc parameter reflects the proportion of skin scales in the image, therefore it can be assumed the lower the SEsc value the less skin scales present in the image and the more hydrated the skin is. This is reflected to some degree in our data, for example the most hydrated skin site was the 5th met. base which has one of the lowest SEsc values.

With regards to the reliability of the elasticity measurements, it has been shown by Bonaparte et al. (2007) that the placement and removal of the Cutometer® probe on the skin between measurement trials does not have a negative impact on reliability [36]. We used this method in this study and found a high level of reliability on all skin sites except for PMA and plantar heel skin sites. The reason for this could be attributed to the relative convex nature of the surface of these sites compared to those of callus for example. Skin in the PMA lies over the bony and joint prominences of the metatarsophalangeal joints and in healthy adults there is a relatively thick layer of subcutaneous connective soft tissue. The heel pad also has subcutaneous fatty tissue to provide cushioning during heel strike. Due to the pliability of this soft tissue it could be that the initial position of the skin within the probe aperture may vary between placements of the probe. Even though the probe has a spring load mechanism to ensure a constant application of pressure to the skin, it may be sensitive to changes in the position or orientation of the soft tissue within the aperture.

The unusually high Reviscometer® error values for the callused skin sites could be attributed to the impacted nature of the tissue. Previous unpublished work by Van Engelen et al. [37] found, by using dermatomed skin grafts over an aluminium plate, the penetration depth of the device to be between 0.5 and 0.7 mm. In thinner grafts, the sound waves reflected off the underlying aluminium plate producing large outlier values. This same phenomenon could be occurring in the case of very hard, callused skin.

With regards to differences in hydration, elasticity, RRT and SESc between different skin sites these data need to be approached with caution due to the small number of participants in each group. However, some promising trends are apparent; in particular the hydration data where high levels of hydration relate to the normal skin sites and the lower values correspond to hyperkeratotic skin (Figs. 3 and 4). Also, the majority (not all) of the differences observed by the measurement devices lie above the SEM for those methods. In order to add confidence to these data further tests would be needed on a larger sample.

## Conclusions

The Corneometer® CM 825, Cutometer® 580 MPA, Visioscan® VC 98 and Visioline® VL 650 Quantiride®, are reliable measurement tools for normal foot skin, xerotic heel skin, heels fissures and plantar callus. It is recommended that SEsc or SEsm measures are used for foot skin as opposed to SEr which has a low level of reliability in this context. The Cutometer has a high sensitivity to herniation of soft tissue into the probe measurement cavity, therefore measurement of plantar soft tissue areas should be approached with caution. The results have also provided error measurements for each method which can be taken into consideration in the design of future studies.

This is the first study to test the reliability of outcome measures of these devices on foot skin in normal and pathological states. The value of these methods is far reaching with regards to their use in quantifying the efficacy of interventions specifically targeted at the treatment of common foot skin conditions.
